# Chiral Carbon Dots as Nanoantennas for Amplification of Molecular Chirality

**DOI:** 10.1021/acsnano.5c22147

**Published:** 2026-03-24

**Authors:** Mateusz Pawlak, Aleksandra Wajda, Zofia Rejman, Maciej Roman, Tomasz P. Wróbel, Agnieszka Kaczor

**Affiliations:** † Faculty of Chemistry, 37799 Jagiellonian University, 2 Gronostajowa St., Krakow 30-387, Poland; ‡ Doctoral School of Exact and Natural Sciences, Jagiellonian University, 11 Lojasiewicza St., Krakow 30-348, Poland; § Laboratory for Biomedical Spectroscopic Applications (LBSA), Faculty of Pharmacy, Jagiellonian University Medical College, 9 Medyczna St., Krakow 30-688, Poland; ∥ SOLARIS National Synchrotron Radiation Centre, Jagiellonian University, 98 Czerwone Maki St., Krakow 30-392, Poland

**Keywords:** nanoscale chirality, carbon dots, chirality transfer, vibrational optical activity, vibrational circular dichroism, nanoparticles

## Abstract

We present herein a previously unreported chiroptical phenomenon, leveraging the intrinsic optical nanoscale-originated properties of chiral ( *l* - and *d* -cysteine derived) carbon dots (C–Dots), specifically, their surface (low-lying electronic excited) states. We show here that these structures generate an exceptional chiroptical response that, furthermore, can be transferred to molecularly chiral entities interacting with C–Dots. In such assemblies, C–Dots behave as nanoantennas, losing their own chiral identity to the advantage of the newly formed chiral (nano)­assemblies that show at least 20-times higher intensity of vibrational optical activity compared to the molecular chirality signal. In a conceptual way, the observed phenomenon is similar to surface-enhanced Raman and Raman optical activity (ROA) spectroscopies, in which nanoparticles, in resonance with the incoming light, enhance the electromagnetic field around analyzed molecules, amplifying their Raman or ROA signal. Our findings enable the application of C–Dots as highly efficient chirality nanosensors which, in light of their rich surface chemistry, translates to the broad potential for the development of C–Dots-based nanoprobes with tailored properties as well as for the rational design of next-generation chiral nanomaterials.

## Graphical abstract

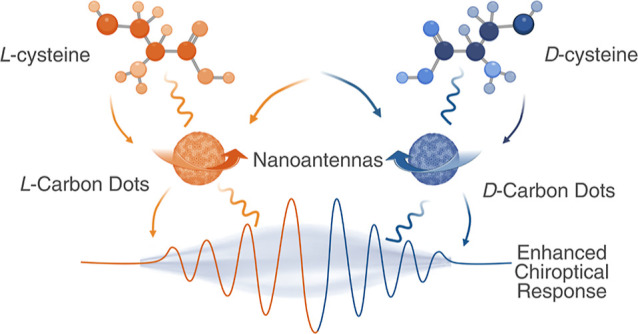

Carbon dots (C–Dots) are an emerging class of carbon nanomaterials that are extensively investigated due to their distinctive optical properties,^1^ in particular tunable and excitation-dependent fluorescence.^2^ Coupled with low toxicity, simple and inexpensive synthesis, high stability, and biocompatibility, these features make them attractive for technological applications.^1,3,4^ The ecocompatibility of C–Dots makes them a greener alternative to semiconductor quantum dots. Therefore, since their serendipitous discovery in 2004,^5^ numerous types of C–Dots have been synthesized, including relatively few chiral ones.^6^ Although the chirality of C–Dots is rather poorly understood, it is evident that it originates from mechanisms that go beyond molecular handedness to nanoscale manifestation.

Nanoscale chirality is gaining significance due to its various applications in chiral catalysis, enantioselective separation and sensing, chiral photonics, as well as data encryption and storage.^7–10^ Nanoscale asymmetric architectures may generate a very intense chiroptical signal, particularly in the vibrational optical activity (VOA). VOA is a newer generation of chiroptical spectroscopies, with exceptional sensitivity to stereostructure, hampered by inherently low analytical sensitivity.^11^ Two manifestations of VOA are vibrational circular dichroism (VCD) and Raman optical activity (ROA), chiral derivatives of infrared absorption and Raman spectroscopy, respectively. As VCD relies on the differential absorption of left and right circularly polarized infrared light by chiral molecules, it is often viewed as an extension of electronic circular dichroism (ECD), which, using light in the UV–vis range, applies the same principle to electronic transitions. However, as a vibrational technique, VCD exhibits higher sensitivity to molecular structure than ECD and may also display distinct sensitivity to nanoscale handedness. It has been shown that nanoscale chirality significantly enhances the VOA response, although to date, only a handful of examples have been reported,^12^ with amyloid fibrils representing one of the most prominent examples showing such enhancement in both VCD^13^ and ROA.^14^

Nanoscale handedness of C–Dots represents a distinct manifestation of quantum size and surface-induced effects^15^ and translates to such phenomena as templating chirality in supramolecular assemblies^16^ or generating chiral (circularly polarized) luminescence.^17^ Chirality of C–Dots can arise from intrinsic core structures with chiral defects or handedness derived from the chiral surface, assembly, or environment.^6^ For C–Dots produced in the bottom-up one-pot synthesis from chiral precursors, it is assumed that chirality originates both from the core and surface states of nanoparticles.^18^ Alternatively, chirality of C–Dots can be introduced as a result of the interaction of achiral surface groups with a chiral environment.^19,20^

Although we are still far from the rational design of C–Dots, there are already a substantial number of synthetic approaches to produce these nanostructures with a wide variety of surface functionalities including hydroxyl, carbonyl, carboxyl, amine, amide, epoxy, sulfonic, and thiol groups. This complex and diverse surface chemistry mediates diverse supramolecular^16^ and covalent^4^ interactions largely responsible for the rapid development of this field of research. This nearly limitless potential of C–Dots for postsynthetic functionalization makes them promising next-generation materials with expanding applications, in which handedness can be strategically employed for chiral recognition and sensing^21–23^ as well as catalytic^24–26^ and nanoenzymatic activity.^27–29^

Herein, by simultaneously exploring the handedness and surface properties of C–Dots, we demonstrate that *l* - and *d* -cysteine-derived carbon nanostructures show characteristic vibrational chiroptical properties originating from their multiple electronic states, which provide distinct conditions for vibronic coupling. It translates into an exceptionally intense nanoscale-derived chiroptical (VOA) signal generated by these structures. The inherent nanoscale handedness can be transferred from C–Dots to compounds exhibiting molecular chirality, as we have shown for cysteine and homocysteine (with an additional gain in the signal intensity). This change in scale provides a significantly enhanced chiroptical output, making C–Dots potent chiral nanosensors that enable efficient chiral recognition and the amplification of molecular chirality.

The complex and diverse chemistry of C–Dots,^4,16^ resulting from a variety of possible functional groups on their surface, may promote the binding of different targets. It translates to the potential for the design of VCD nanosensors with tailored properties, providing a basis for the application of C–Dots for efficient analysis of molecular chiral systems, and may become a basis for the development of next-generation materials with substantial chiral activity.

## Results and Discussion

### Enhanced Vibrational Optical Activity (VOA) Signal of *l* / *d* -Cysteine-Derived Carbon Dots

The synthesis of chiral C–Dots was carried out using radical-assisted synthesis at room temperature^30^ with slight modifications. The chiral properties of C–Dots obtained from *l* - and *d* -cysteine (Scheme 1) are shown in Figure 1.

**Scheme 1:**
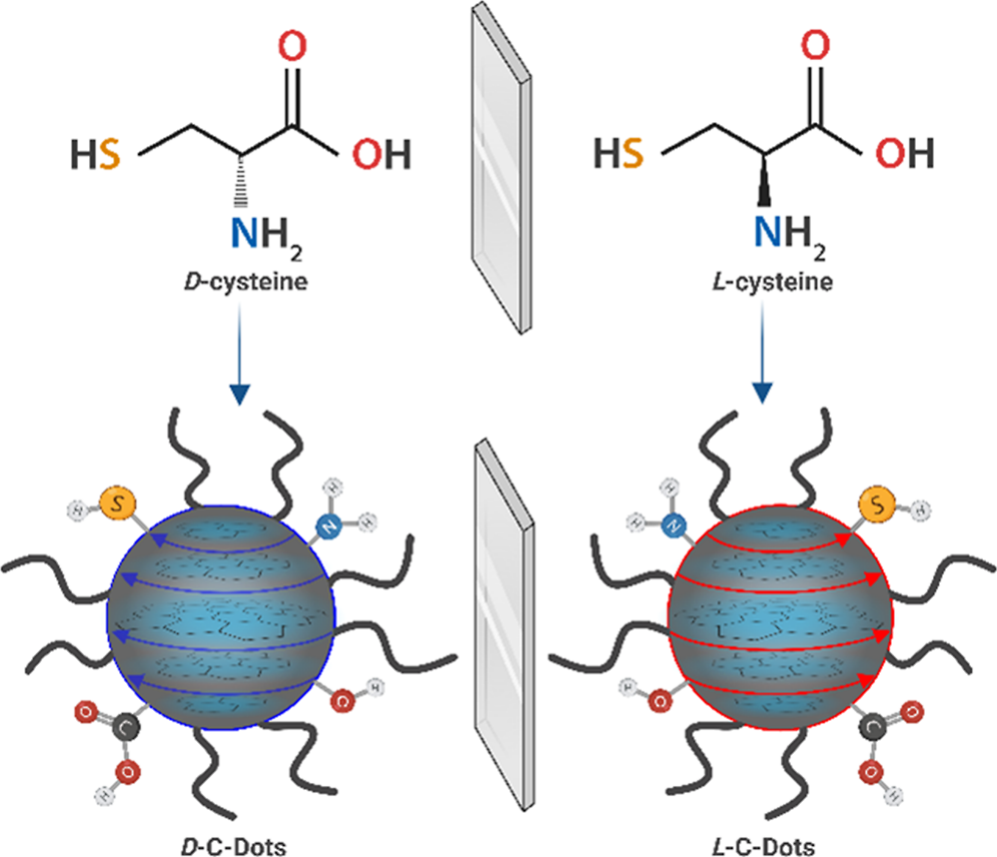
Schematic Representation of *l* - and *d* -cysteine-derived C–Dots

**Figure 1:**
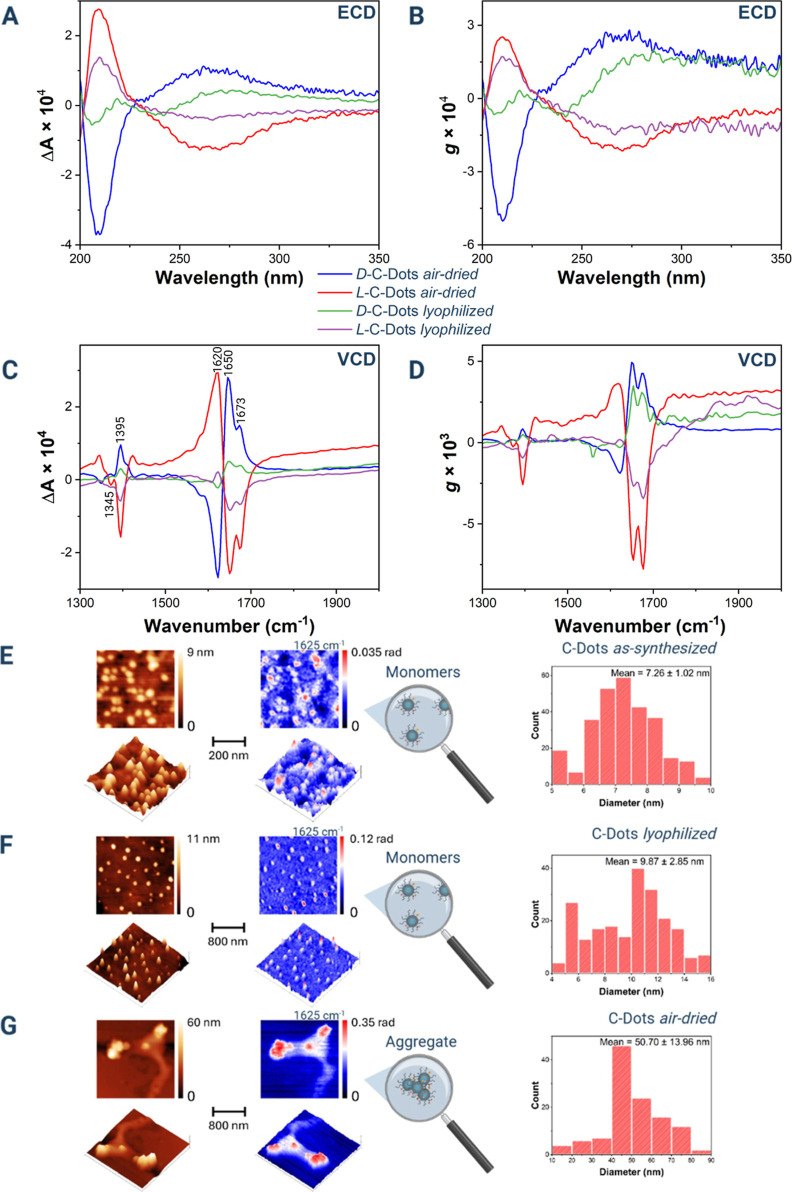
Chiroptical and morphological characterization of *l* / *d* -cysteine-derived C–Dots. Representative ECD (A, 1 mg/mL) and VCD (C, 10 mg/mL) spectra of either air-dried or lyophilized C–Dots after resuspension in D_2_O. The quantitative characteristics of ECD (A) and VCD (C) signal intensity, expressed as the anisotropy factor g for ECD and VCD spectra, are presented in panels B and D. Representative AFM and corresponding *s*-SNOM images (1625 cm^–1^) of as-synthesized (E), lyophilized and resuspended in water (F), or air-dried and resuspended in water (G) C–Dots drop-cast onto a silicon substrate. Respective electronic absorption and infrared spectra are provided in Figures S1 and S2. Size distribution analysis was based on AFM images, using 286, 216, and 117 structures for as-synthesized, lyophilized, and air-dried samples, respectively.

Similarly to other supramolecular systems studied previously,^12,31^ ECD poorly reflects the nanoscale chirality of C–Dots. This is evidenced by the lower ECD intensity of cysteine-derived C–Dots compared to ECD of cysteine enantiomers measured under the same conditions (Figures 1A and S1C), resulting in approximately 10 times lower anisotropy factor g_ECD_ for cysteine-derived C–Dots compared to g_ECD_ of cysteine itself. The anisotropy factor is a quantitative measure of the CD response of a system. It is the ratio of the CD signal to the respective absorption signal at the same wavelength (or for its range) and can be defined as

$$g=\frac{\Delta A}{A}=\frac{\Delta \varepsilon }{\varepsilon }$$

where A and ε are absorption and molar absorption coefficient, respectively, and ΔA and Δε are differences in absorption and molar absorption coefficients, respectively, for left and right circularly polarized light.

Typically, g_ECD_ does not exceed 10^–2^, and for cysteine-derived C–Dots, it exhibits a value in the 10^–4^ range (Figure 1B), confirming that nanoscale chirality does not significantly contribute to the ECD response.

A very different situation is observed for VCD. The typical g_VCD_ values are in the 10^–4^–10^–5^ range, hence 2–3 orders of magnitude weaker than g_ECD_. Thus, in contrast to ECD, VCD of C–Dots derived from *l* - and *d* -cysteine is exceptionally intense (Figures 1C and S2A). The anisotropy factor g_VCD_ approaches 10^–2^ for C–Dots after air-drying and resuspension in D_2_O and equals ca. 4 × 10^–3^ if nanostructures are first lyophilized and then resuspended (Figure 1D). It demonstrates that VCD of C–Dots is nearly 2 orders of magnitude larger compared to the “reference” value of 10^–4^ (and the g_VCD_ value for cysteine that is in this range, Figure S2C). To date, only one study demonstrated VCD of amyloid fibrils derived from either (*R*,*R*)- or (*S*,*S*)-1,2-cyclohexanediamine and arginine,^32^ yet it has not reported its amplification, showing that it is not simply derived from the nanoscale chirality. We address below the mechanisms explaining the signal amplification that we observe, its further unanticipated enhancement upon the binding of molecularly chiral entities, and the consequences of the observed phenomena.

### Aggregation Is not the Key Factor Determining the Enhanced VCD Response of Cysteine-Derived Carbon Dots

The most intense VCD band, a couplet, with the extrema at 1650 (shoulder at 1673) and 1620 cm^–1^, originates from the asymmetric stretching vibrations of the surface COO– groups of C–Dots. The band is shifted toward higher wavenumbers relative to this VCD band for cysteine in D_2_O observed at ca. 1620 cm^–1^, as a result of interactions with the carbon cores of the nanostructures. The lower-intensity signals at ca. 1395 and 1345 cm^–1^ are due to the COO– symmetric stretching and CH bending vibrations.^33^ For statistical characterization of C-dots’ size, scattering type scanning near-field optical microscopy (*s*-SNOM), together with atomic force microscopy (AFM) (Figures 1E–G and S3), were employed as *s*-SNOM is more informative in the context of functional groups than typically used transmission electron microscopy (TEM). However, TEM was applied to visualize a carbon core of the studied structures, indicating the graphene interlayer distance of approximately 0.32 nm (Figure S4). The comparison of AFM images and the respective *s*-SNOM images showing the distribution of the carbonyl groups demonstrates the significant abundance of these functionalities in cysteine-derived carbon nanostructures (Figures 1E–G and S3). Both VCD and *s*-SNOM indicate that for the studied C–Dots, they constitute dominant surface groups, but the presence of another functional group is also demonstrated by ATR-IR spectra of solid C–Dots (Figure S5), in particular –SH and –NH_2_ moieties, as evidenced by their bands at 2588 cm^–1^ (–SH) and 3300, 3240, 3120 cm^–1^ (−NH_2_).

The essential observation emerging from high-resolution microscopic characteristics combined with VCD is the difference in the sizes of as-synthesized C–Dots and C–Dots that are subjected to air-drying and then dispersed in a solvent.

*s*-SNOM/AFM show clear evidence of aggregation of these nanostructures when solvent-dispersed (Figure 1G). As-synthesized nanoparticles are monomeric (Figure 1E) of typical heights below 10 nm (an average diameter of ca. 7.3 nm), and nanostructures obtained by air-drying and resuspension in D_2_O are characterized by a significantly larger diameter with an average of approximately 50.7 nm. Aggregation-induced enhancement, where aggregates yield stronger signals than monomers, is well established, particularly in fluorescence as Aggregation-Induced Emission (AIE).^34^ It was also previously recognized for ECD and VOA (both VCD and ROA) as a result of exciton chirality.^35^ Thus, to verify if aggregation contributes to the observed VCD signal enhancement, we measured the VCD response of C–Dots lyophilized and resuspended in D_2_O. This step was necessary because quantitative evaluation of the anisotropy factor g_VCD_ for as-synthesized C–Dots is precluded by dominating water (H_2_O) signals that introduce artifacts. Figure 1F confirms that this procedure prevents aggregation (the average diameter of ca. 9.9 nm), simultaneously resulting in a ca. 2-fold decrease of g_VCD_ compared to aggregated C–Dots (Figure 1D). However, the signal of monomeric (lyophilized and resuspended) C–Dots is itself enhanced with g_VCD_ of ca. 4 × 10^–3^. This demonstrates that self-assembly of cysteine-derived C–Dots, although it may slightly contribute to signal increase, is not the key source of the observed amplification.

Instead, the key mechanism of amplification of the chiral readout is related to the inherent structural features of these nanoparticles, as is shown below.

### Low-Lying Surface States of Carbon Dots Enable Vibronic Coupling That Is a Key Mechanism Contributing to Their Enhanced VCD

Among the numerous particular optical properties of C–Dots, excitation-dependent emission is arguably the most extensively studied.^1,2^ This phenomenon arises from the existence of numerous electronic states within the C–Dots, covering a broad spectral range from the visible to the near-infrared. The UV–vis–NIR spectrum demonstrates a continuous absorption tail extending to at least 1600 nm (6250 cm^–1^), confirming the presence of low-lying electronic states (LLES) (Figure S6). Existence of multiple electronic states is further evidenced by fluorescence (Figure S7).

Although the fluorescence mechanism of C–Dots is not fully elucidated, it is established to stem from quantum confinement effects of conjugated π-domains, surface/edge states, or a combination of both factors.^18,36,37^ This implies that the fluorescence of C–Dots can be tuned both by the size of these nanostructures and via modifications of their surface chemistry.^36^ The surface states are low-lying excited electronic states related directly to functional groups of C–Dots (Figure 2). We identify this inherent feature of C–Dots—specifically, the presence of LLES—as the key factor enabling the observed enhancement of the VCD signal. A recognized mechanism for VOA signal enhancement involves the coupling of excitation energy to the energy of electronic transitions. In the case of ROA, a considerable impact of electronic resonance on signal intensity has been demonstrated for numerous molecules and supramolecular systems using excitation in the visible range.^38–47^ For VCD, such an effect is less common, as its prerequisites are low-lying electronic excited states. The vibronic theory of VCD incorporating LLES was developed by Nafie.^48^ Several studies have experimentally demonstrated vibronic-LLES coupling, primarily in open-shell metal complexes possessing low-lying d and f states,^49–51^ a topic recently systematically reviewed by Pescitelli and Di Bari.^52^ Given that VCD signal enhancement via vibronic coupling is well documented for metal complexes, a plausible explanation for the highly intense VCD observed in C–Dots is an analogous phenomenon resulting from the coupling of vibrational states with surface states (LLES) in these structures (Figure 2). Hence, the significant intensity and selectivity of the VCD signal can be explained by the effective coupling of the modes in the 1670–1620 cm^–1^ range (assigned to C=O stretching vibrations, possibly with some contribution from C=N and C=C modes) with these LLES, which generates a significant magnetic dipole moment via charge circulation. The magnitude of the effect observed for C–Dots is consistent with the enhancement identified for metal complexes. As demonstrated in Figure 1D, this effect is responsible for a 10- to 20-fold amplification of the VCD signal (ca. 4 × 10^–3^ for monomeric C–Dots) compared with the typical “reference” VCD intensity (ca. 1 × 10^–4^). It agrees well with the previously reported values for open-shell metal complexes, which typically exhibit bands 1 order of magnitude (10–20 times) more intense than isostructural complexes with closed-shell metal ions.^49–51^ Although the facts presented above strongly suggest that vibronic coupling is the mechanism behind the observed VCD enhancement, further research is needed to explain the nature of LLES in C–Dots.

**Figure 2:**
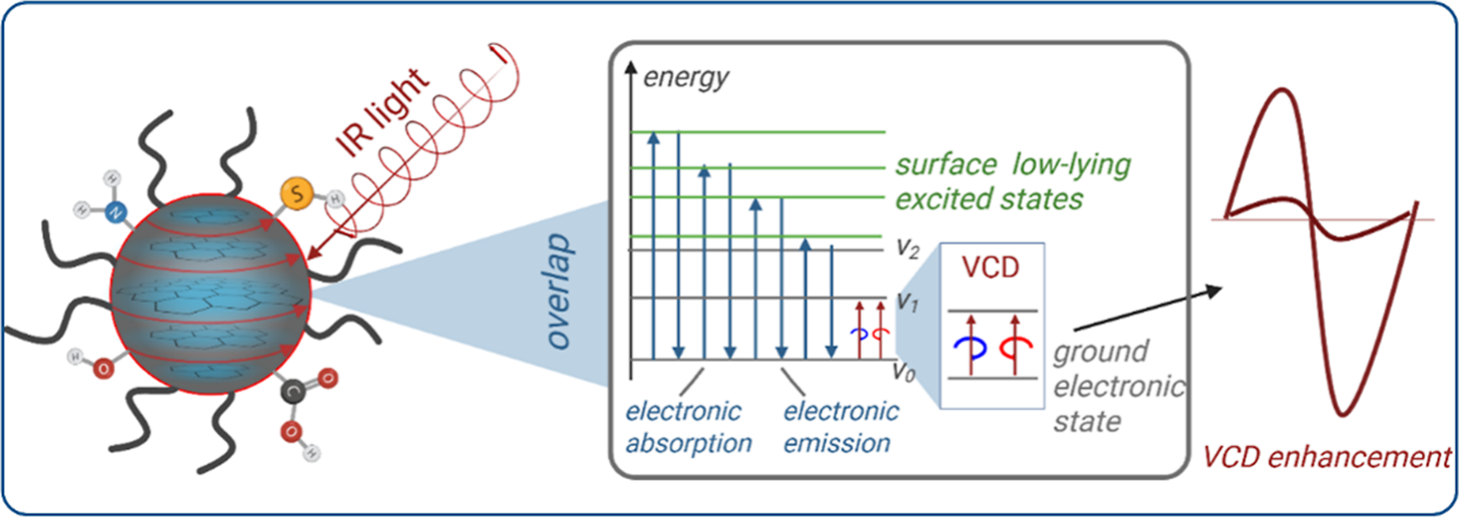
Vibronic mechanism of VCD signal enhancement in C–Dots. Nanostructures as C–Dots are characterized by energy confinement occurring due to spatial restrictions on the movement of electrons that leads to multiple discrete electronic states. Low-lying electronic states are controlled by the surface groups of the C–Dots. Vibronic coupling of the surface electronic states and the vibrational states generates the enhancement of the VCD signal, as shown by the anisotropy factors g_VCD_ in the 4 × 10^–3^–8 × 10^–3^ range.

Although the exceptionally intense VCD of C–Dots is clearly interesting per se, more importantly—as elaborated below—it may allow for using these structures as tunable and efficient VCD nanosensors of molecular chirality.

### Nanoscale Transfer of Enhanced Chiral Signal Proves Carbon Dots to be Exceptionally Efficient Nanosensors of Molecular Chirality

Amino acids show a weak VCD signal, making them difficult analytes for VCD measurements in solution. The VCD of *l* - and *d* -cysteine, presented in Figure 3A (and Figure S2C), and recorded at a concentration of 14 mg/mL with the total acquisition time of 600 min, show low-intensity signals: a band at ca. 1620 cm^–1^ due to the COO^–^ asymmetric stretching vibrations and a signal at 1340 cm^–1^, assigned to the CH bending mode.^33^ The addition of C–Dots to the cysteine solution (in a 1:1 mass ratio, 10 mg/mL each) initially results in the appearance of the characteristic VCD of C–Dots; however, this signal undergoes significant changes over time. The time evolution of the VCD signal is shown in Figure 3C.

**Figure 3:**
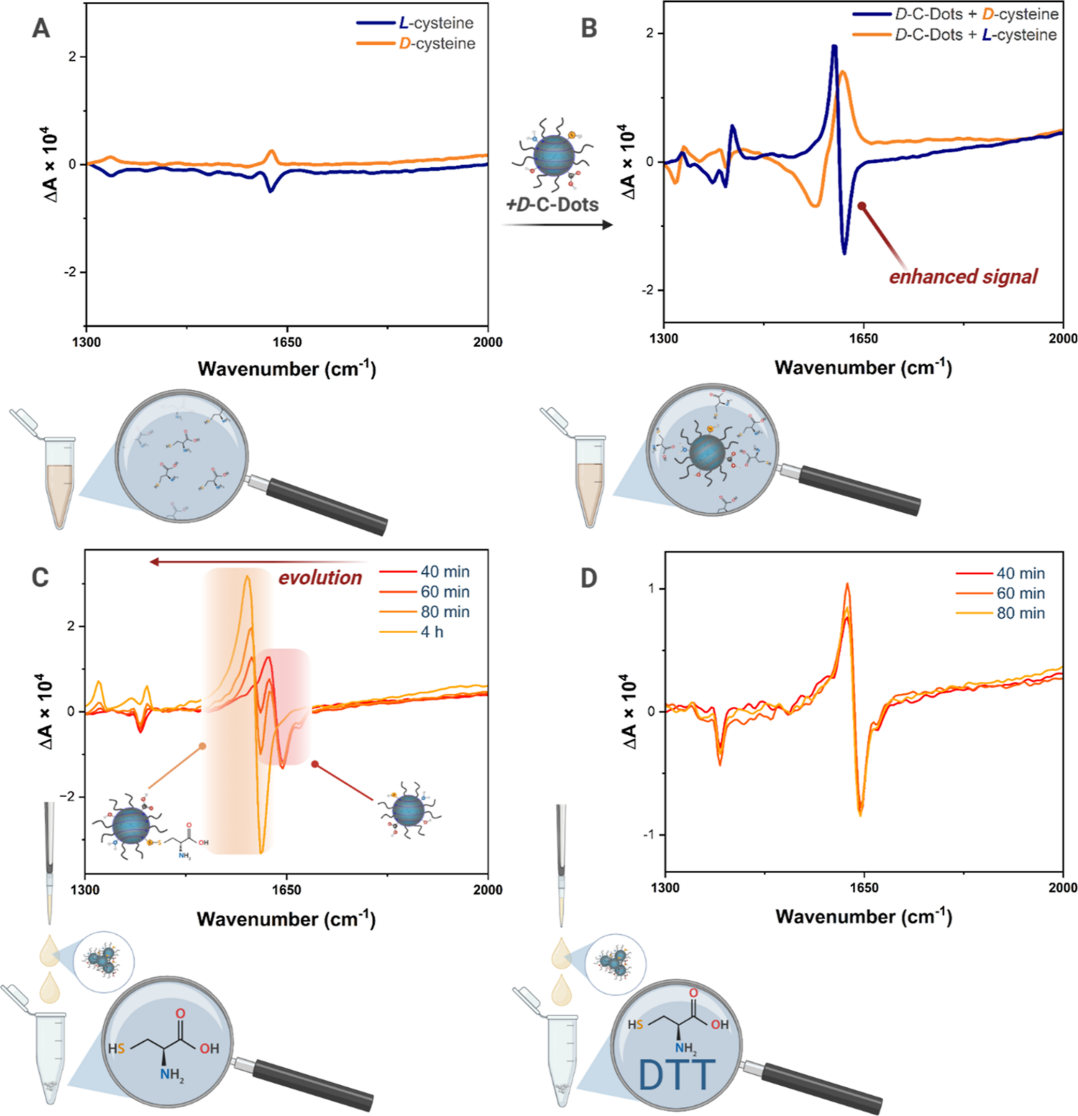
Transfer of nanoscale vibronically enhanced chirality of C–Dots to molecular entities. VCD spectra of cysteine in D_2_O (A, total registration time 600 min per spectrum, concentration of 14 mg/mL, path length 15 μm) show low intensity signal with g_VCD_ in the 10^–4^ range. Postfunctionalization of C–Dots with cysteine demonstrates that the intense nanoscale vibronically coupled signal is transferred to cysteine; hence *l* and *d* enantiomers of cysteine show almost mirror image VCD spectra (B, D_2_O, total registration time 20 min per spectrum, concentration of both reactants equal to 10 mg/mL, path length 50 μm). The reaction between C–Dots and cysteine is completed after 4 h (C), as shown by a complete disappearance of the C–Dots’ signal. The reaction of C–Dots and cysteine leading to the formation of disulfide bonds is blocked by dithiothreitol (DTT, a classical reducing agent that cleaves disulfide bonds), as evidenced by the characteristic VCD spectra of C–Dots under these conditions (D). The respective infrared spectra are provided in Figures S2D and S8, for VCD shown in panels A and B–D, respectively.

Specifically, the intensity of the signal originating from C–Dots begins to decrease, and 40 min after the reaction initiation, traces of a new couplet at ca. 1610/1570 cm^–1^ are observed. The reaction is complete after 4 h (Figure 3B,C), as evidenced by the total disappearance of the original C–Dots signal, the formation of the new couplet at 1610/1570 cm^–1^, and simultaneous considerable changes of the spectral shape in the lower wavenumber range. Interestingly, this newly generated signal is further amplified compared to the already intense signal of C–Dots. The main signals in the spectra recorded for the respective diastereoisomeric pairs ( *l* -C–Dots with the *l* -and *d* -cysteine pair as well as *d* -C–Dots with the *l* -/ *d* -cysteine pair, Figure 4) are nearly mirror images. This indicates that the nanostructures provide enantiorecognition of *l* - and *d* -amino acids, independent of the native chirality of the C–Dots (although subtle differences in the positions of the newly formed couplet are observed for *l* - and *d* -C–Dots, Figure 4). Thus, postfunctionalization of C–Dots with molecularly chiral entities triggers nanoparticles to lose their own chiral signature, which implies that they start to act as nanoantennas, significantly amplifying the molecular chirality of the attached chiral entitles. Taking into account the anisotropy factor g_VCD_ of ca. 10^–4^ for cysteine, the enhancement of the VCD output via C–Dots is at least 20-fold. Intriguingly, the ECD signal of the postfunctionalized C–Dots is also approximately four times more intense (Figure S10) compared to the signal of C–Dots alone. It shows that C–Dots can function as a platform for enhancement of the chiroptical signal, which is particularly important for VOA.

**Figure 4:**
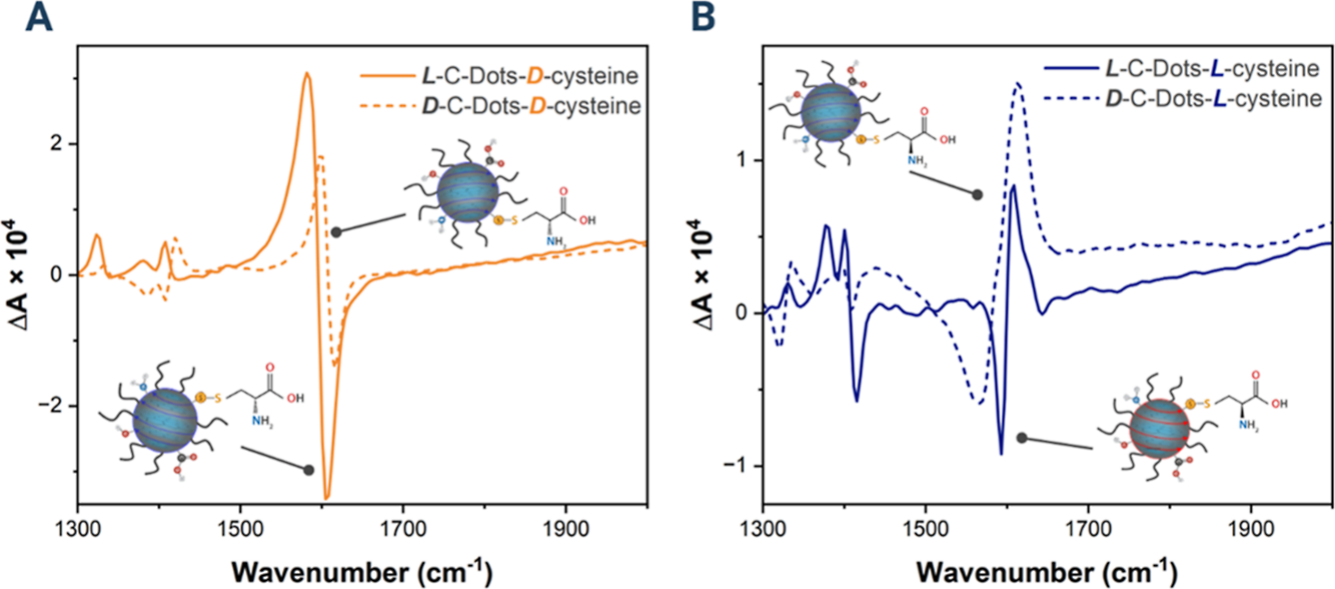
Postfunctionalization of C–Dots with molecularly chiral entities (cysteine) triggers them to lose their own chiral signature. The signal of C–Dots- *d* -cysteine (A) shows the same signs of the main couplet independently on the C–Dots original chirality; the same is observed for C–Dots- *l* -cysteine. (B) Signals of *D*- and *l* -cysteine are quasi mirror images in pairs: *D*-C–Dots-*D*- and *l* -cysteine as well as *L*-C–Dots-*D*- and *l* -cysteine showing that the molecular handedness (amplified by C–Dots) is the origin of the chiroptical signal identity. Respective infrared spectra are provided in Figure S9.

To elucidate the mechanism of the reaction between C–Dots and cysteine, we performed several additional experiments. Mixing C–Dots with other amino acids (phenylalanine, tyrosine, serine, and alanine) using an analogous procedure did not result in any change to the original VCD signal, preserving the signature of C–Dots (Figure S11); however, the reaction with homocysteine induced a new chiroptical signal (Figure S12). Consistent with this finding, the reaction of C–Dots with cysteine in the presence of dithiothreitol (DTT; in a 1:1:1 mass ratio relative to C–Dots and cysteine, 10 mg/mL each)—which acts as a reducing agent by cleaving disulfide bonds—also did not produce any change in the original VCD signal (Figure 3D). Based on these observations, it appears that the reaction between cysteine-derived C–Dots and cysteine results from spontaneous disulfide bond formation at room temperature. The kinetics of the process also provide confirming evidence, as the reaction takes ca. 4 h, i.e., it is rather slow at room temperature, in agreement with typical S–S bond formation times.^53^ After this time, the VCD signal is derived (at least predominantly) from the C–Dots-cysteine system that generates a different VCD signal from unsubstituted C–Dots. Crucially, its chirality is controlled by the handedness of the attached cysteine enantiomer (and not the chirality of C–Dots). The formation of disulfide bonds, which covalently link cysteine and C–Dots, induces the transfer of molecular chirality to the nanoscale level. This transfer, facilitated by a vibronic coupling mechanism, provides the observed enhancement of the chiral (vibrational optical activity) signal. Binding of the ligand to C–Dots results in a red shift of the COO^–^ stretching vibrations to 1610/1570 cm^–1^ or 1610/1600 cm^–1^ for *l* - and *d* -C–Dots-cysteine assemblies, respectively. Together with the lack of predominant influence of handedness of C–Dots on the observed VCD signature, it highlights their role as efficient nanosensors, transferring nanoscale chirality to the analyte.

## Conclusions

The reported optical phenomenon of chiroptical signal amplification via C–Dots provides increased capabilities for chiral research. The very origin of this VCD enhancement lies in the same mechanism responsible for excitation-dependent fluorescence: i.e., quantum confinement within the nanoscale domains, leading to multiple discrete energy levels that manifest in both absorption and emission spectra. Specifically, the VCD signal in C–Dots is enhanced by vibronic coupling between vibrational states and low-lying excited (surface) states—a feature inherent to C–Dots in general, rather than being specific to those derived from cysteine. This underscores the broad relevance of our findings; it implies that whenever rational synthesis is employed to design C–Dots with surface chemistry tailored for binding a specific molecule, amplification of that entity’s molecular chirality can be achieved.

The exceptional intensity of VCD of C–Dots offers a capability for tracking the reaction progress, the reaction mechanisms, etc. Yet, first and foremost, it provides a route for using chiral C–Dots as universal chiral nanosensors for amplification of molecular chirality. In a conceptual way, the observed phenomenon is similar to surface-enhanced Raman^54^ and in particular surface-enhanced ROA^45^ ^,^ ^55–57^ spectroscopy, where nanoparticles, in resonance with the incoming light, enhance the electromagnetic field around the analyzed molecules, amplifying their Raman or ROA signal. While the amplification in the studied C–Dots is mediated via covalent (disulfide, S–S) bonds, we anticipate the future discovery of related phenomena. These may include the enhancement of molecular chirality via achiral C–Dots, the induction of chiroptical signals in achiral molecules through the interaction with chiral C–Dots, and amplification resulting from supramolecular binding between ligands and nanostructures.

Even in the unlikely scenario that this phenomenon is limited to covalently mediated assemblies, it offers promising opportunities for the efficient and enantioselective analysis of chiral systems and serves as a foundation for the development of next-generation materials with pronounced optical activity. Ultimately, this phenomenon may be recognized as universal, likely occurring not only in C–Dots but also in a wide array of nanostructures whose properties are governed by the combination of electron confinement and surface effects (e.g., quantum dots, perovskites, nanowires). In this context, our findings may help to uncover other optochiral phenomena relevant to various spectroscopic techniques.

## Methods

### Materials

*l* -Cysteine (98.5% or 99%), *l* -homocysteine (98%), *l* -alanine (98.5%), *l* -phenylalanine (98.5%), *l* -serine (99%), *d* -tyrosine (99%), sodium hydroxide (98%), and copper­(II) acetate (99%) were purchased from Merck. 35–37% hydrochloric acid was purchased from Chempur. *d* -Cysteine (99%) was purchased from Thermo Fisher. Ultrapure (Millipore) water was used. For purification, a 1000 Da dialysis membrane (SpectraPor) was employed. The ECD and VCD measurements were conducted in D_2_O (99.98%, Merck).

### Preparation of C–Dots

The synthesis of chiral C–Dots was carried out using radical-assisted synthesis at room temperature^30^ with slight modifications (Scheme 2). Briefly, 150 mg of *l* - or *d* -cysteine (1.24 mmol) was dissolved in 100 mL of Millipore water, and the pH was adjusted to 10.5 using freshly prepared 2 M NaOH. Then, to induce the radical-assisted formation of C–Dots, 0.200 mL of 0.25 M Cu­(OAc)_2_ (50 μmol) was added under vigorous stirring, and the reaction mixture was stirred at room temperature for 2 h. The reaction was quenched by acidifying the solution (pH = 7) with a previously prepared 2 M HCl solution. The precipitated byproduct was removed by centrifugation (10,000 rpm, 5 min) using an MPW-260RH centrifuge. The supernatant was purified by dialysis against 2.5 L of distilled water for 24 h, and the product was obtained by air-drying at room temperature, resulting in approximately 20 mg of a black powder. Corresponding samples prepared in the same conditions were lyophilized for 48 h, resulting in approximately 10 mg of an extremely lightweight, airborne powder.

**Scheme 2:**
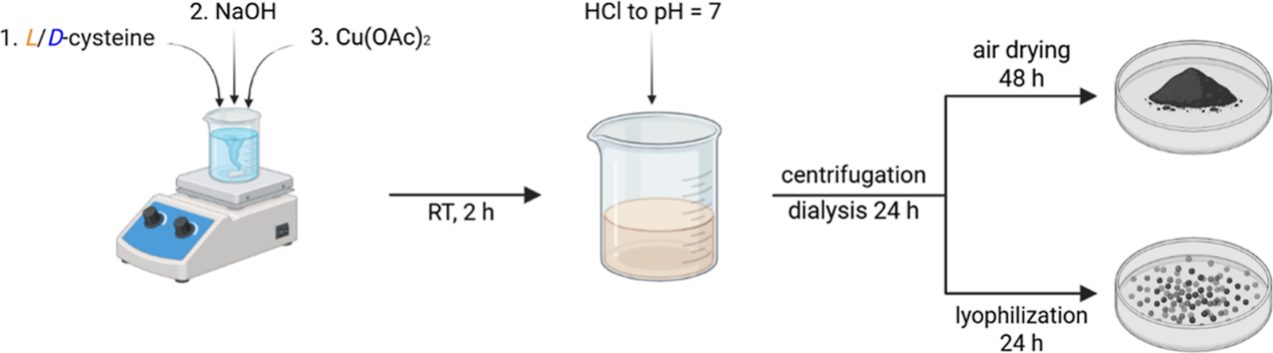
Diagram Illustrating the Main Steps Involved in the Preparation of C-Dots

### C–Dots Assemblies with Cysteine and Homocysteine

2 mg of enantiomerically pure crystalline *l* - or *d* -cysteine was placed into four individual 1.5 mL Eppendorf tubes. Then, 200 μL of a freshly prepared 10 mg/mL solution of *L*- or *D*-C–Dots in D_2_O was added to each sample, resulting in four distinct chiral combinations. The mixtures were sonicated for 15 min using a Pulsonic 05 ultrasonic bath. After sonication, the samples were directly used for VCD measurements. The progress of chirality transfer between *l* - and *d* -C–Dots and cysteine ( *l* and *d* enantiomers) was monitored by observing changes in the VCD spectral bands over time. Once spectral stabilization was observed—indicating the completion of the chiral transfer process—the samples were diluted prior to ECD analysis. Each sample was first diluted 10-fold and then further diluted by two-thirds, resulting in a final concentration of approximately 0.66 mg/mL. The resulting solutions were used for ECD measurements. The samples for reaction with homocysteine were prepared in a similar way but in the weight ratio of 1:4, using 100 μL 10 mg/mL solution of *d* -C–Dots in D_2_O, which was added to 2 mg of homocysteine, mixed vigorously, and sonicated as described above.

### C–Dots Assemblies with Other Amino Acids

To check the possible interaction with other amino acids, a series of experiments was conducted. To 2 mg of *l* -serine, *d* -tyrosine, *l* -phenylalanine, or *l* -alanine, 200 μL of 10 mg/mL *l* -C–Dots solution was added. The prepared samples were sonicated for 15 min in an ultrasound bath. Then, the mixture was poured into the CaF_2_ cuvette with the 50 μm spacer and measured using VCD at room temperature.

### UV–Vis–NIR Electronic Absorbance

UV–Vis–NIR absorption spectra of C–Dots were recorded in the 200–1600 nm range. A D_2_O suspension of C–Dots at a concentration of 0.1 mg mL^–1^ was used for measurements over 200–1600 nm, and a concentration of 1 mg mL^–1^ was used for measurements in the 400–1600 nm region. The pre-prepared C–Dots suspensions were sonicated for 15 min prior to each measurement. Spectra were recorded using a Shimadzu UV-360i Plus spectrophotometer with a 10 mm path length quartz cuvette. An integrating sphere was employed to minimize the contribution of scattering to the measured extinction, confirming that the observed tail up to 1600 nm originates from absorption. Measurements were performed with a scanning speed of 100 nm/min, a spectral bandwidth of 1 nm, and a data pitch of 0.2 nm.

### Fluorescence

For fluorescence measurements, a D_2_O suspension of C–Dots was prepared at a concentration of 1 × 10^–4^ mg mL^–1^. The suspension was sonicated for 15 min prior to each measurement. Spectra were recorded using a Jasco J-1500 spectrometer equipped with a fluorescence accessory and a quartz cuvette with a path length of 3 mm. Emission spectra were collected at excitation wavelengths ranging from 280 to 420 nm in 20 nm intervals, with a scanning speed of 100 nm min^–1^, a bandwidth of 6 nm, and a data pitch of 1 nm.

### Electronic Circular Dichroism (ECD)

For electronic absorption and ECD analysis, an aqueous solution of C–Dots was prepared in D_2_O at a final concentration of 1 mg/mL. This solution was obtained by a 10-fold dilution of the C–Dots dispersion initially prepared for VCD measurements. Before dilution, the C–Dots were subjected to ultrasonic treatment using a Sonix VCX 130A-200 probe-type sonicator for 15 min to ensure full dispersion and prevent aggregation. The resulting suspension was visually homogeneous and was immediately used for spectral analysis. The spectra were recorded by using a Jasco J-1500 spectrometer in a quartz cuvette with a 1 mm path length. Measurements were performed in the range of 200–350 nm, with a scanning speed of 50 nm/min, a bandwidth of 1 nm, and a data pitch of 0.2 nm. For the samples described in the C–Dots assemblies with cysteine and homocysteine section, the postsonication mixtures were further diluted to achieve a final concentration of approximately 0.66 mg/mL for the combination of C–Dots with cysteine and 0.5 mg/mL for *d* -C–Dots with *l* -homocysteine. These samples were also subjected to ECD analysis under the same instrumental parameters.

### Vibrational Circular Dichroism (VCD)

FTIR and VCD spectra were measured using a BioTools ChiralIR-2X spectrometer equipped with DualPEM technology. Before measurements, C–Dots samples were sonicated for 15 min using a Sonix VCX 130A-200 sonicator with the following settings: amplitude = 45%, pulsed mode with 5 s of sonication followed by 25 s of pause to avoid sample overheating. Then, 75 μL of C–Dots solution at a concentration of 10 mg/mL in D_2_O was placed in a CaF_2_ cuvette with a path length of 50 μm. Spectra were collected at room temperature for approximately 1.5 h with a spectral resolution of 8 cm^–1^. VCD spectra of *l* / *d* -cysteine were obtained from a saturated solution in D_2_O, diluted twice, in a BaF_2_ cuvette with a path length of 15 μm and spectral resolution of 8 cm^–1^. Samples described in the C–Dots assemblies with cysteine and homocysteine sections were measured under the same conditions as C–Dots, but for a prolonged time of 7 h to monitor the reaction progress. The spectral resolution for the initial measurement was 8 cm^–1^.

### ATR-FTIR

ATR-FTIR spectra were recorded by using a Thermo Scientific Nicolet iS5 FTIR spectrometer equipped with a diamond-attenuated total reflectance (ATR) crystal. Before each sample measurement, a background spectrum was collected under identical instrumental conditions to account for atmospheric and instrumental contributions and to enable an accurate baseline correction. For analysis, samples of *l* - and *d* -C–Dots were placed directly onto the surface of the diamond crystal. The samples were then gently but firmly pressed against the crystal using the built-in pressure arm of the ATR accessory to ensure uniform and stable contact, which is essential for reliable signal acquisition. The ATR crystal was cleaned with ethanol prior to each measurement to remove any residual materials and prevent cross-contamination between the samples. Spectra were acquired at room temperature, using a spectral resolution of 8 cm^–1^, and each final spectrum was generated by averaging 32 consecutive scans to improve the signal-to-noise ratio. All measurements were made under ambient laboratory conditions.

### AFM/*s*-SNOM Measurements

AFM and *s*-SNOM images were recorded at the SOLARIS National Synchrotron Radiation Centre in Krakow using a neaSCOPE microscope (attocube systems AG, Germany) equipped with a quantum cascade laser (QCL), a high NA (0.46) parabolic mirror, and a liquid-nitrogen-cooled MCT IR detector. The measurements were carried out in a tapping mode using ARROW-NCPt tips (NanoWorld, Switzerland) with an apex radius of 30–40 nm. Aqueous solutions of *l* / *d* -C–Dots at a concentration of 1 mg/mL were diluted approximately 100 times and subsequently drop-cast onto a silicon substrate. After solvent evaporation, the sample-coated substrates were placed in a measurement chamber. The images were acquired at 1625 cm^–1^ with 1 mW laser power. *s*-SNOM images represent background-free phase signals demodulated in the second harmonic of the AFM cantilever mechanical resonance frequency (φ_2_). All images are normalized (i.e., referenced) to the phase value of the Si reference substrate (φ_2_ = φ_2,sample_ – φ_2,reference_). The images were processed (background subtraction, artifact corrections – scars, and shifts of *x*-axis) using Gwyddion software (Czech Metrology Institute, Czech Republic).

### TEM Measurements

TEM imaging was carried out with a Tecnai Osiris instrument (FEI) with an X-FEG Schottky field emitter operating at an accelerating voltage of 200 kV and a Rio16 camera (Gatan). The samples were deposited on a lacey carbon film supported on a copper grid (Agar Scientific, 400 mesh). Before microscopic examination, the *l* / *d* -C–Dots aqueous solutions (1 mg/mL) were diluted 100 times. Subsequently, the prepared solutions were deposited onto copper grids coated with a carbon film (Lacey Carbon Film, 300 Mesh Cu, Agar Scientific). The prepared grids were counterstained using UranyLess staining solution (UranyLess EM Stain, 30 mL airless bottle).

### Spectra Processing

Electronic absorption and ECD spectra were analyzed using JASCO version 1.52.00 [Build 4] Copyright JASCO Corporation 1995–2000, OPUS 7.2 [Build: 7, 2, 139, 1294] Copyright Bruker Optik GmbH 2012. FTIR and VCD spectra were processed in OPUS 7.2 [Build: 7, 2, 139, 1294] Copyright Bruker Optik GmbH 2012. All spectra were analyzed with OriginPro 2021b 9.8.5.201 (Academic) Copyright 1991–2021 OriginLab Corporation 1991–2021 software. The ECD, electronic absorption, infrared, and VCD spectra were background-corrected by using the spectra of the solvents obtained under the same conditions. For the VCD spectrum of *l* -cysteine, the baseline (a straight-line) correction was used to account for the baseline inadequacies. Spectral offset was used if necessary.

## Supplementary Material



## Data Availability

Additional data sets generated and/or analyzed during the current study are available from the corresponding author on reasonable request. The preprint is posted on ChemRxiv [DOI:10.26434/chemrxiv-2025-p9wkb].

## Notes

Additional images and spectra: ECD and electronic absorption spectra of air-dried or lyophilized C–Dots and cysteine, VCD and infrared spectra of air-dried and lyophilized C–Dots and cysteine, representative AFM and corresponding *s*-SNOM images of as-synthesized, lyophilized, or air-dried C–Dots, representative TEM images of C–Dots sonicated for 15 min and without sonication, ATR-IR spectra of solid C–Dots and cysteine, absorbance spectra of air-dried *L*/*D*-C–Dots, fluorescence spectra of *L*-C–Dots and *D*-C–Dots, VCD and IR spectra of C–Dots postfunctionalized with *L*/ *d* -cysteine, time-dependent changes, and spectra in the presence of dithiothreitol (DTT), VCD and IR spectra of C–Dots with *d* -cysteine and *l* -cysteine, ECD and electronic absorption spectra of *d* -cysteine-derived C–Dots and *l* -cysteine-derived C–Dots, VCD and IR spectra of *L*-C–Dots combined with *l* -phenylalanine, *l* -alanine, *l* -serine, and *d* -tyrosine, and VCD, IR, ECD, and electronic absorption spectra of *L*-C–Dots combined with *l* -homocysteine (PDF)
