# Supplementary material for: Chiral Carbon Dots as Nanoantennas for Amplification of Molecular Chirality
Source: ACS Nano. 2026 Mar 24;20(14):11219–29. doi: 10.1021/acsnano.5c22147 (PMC13299002; doi:10.1021/acsnano.5c22147)
Supplement: Supplementary file 1 [file nn5c22147_si_001.pdf]

# Electronic Supplementary Materials

## Chiral Carbon Dots as Nanoantennas for Amplification of Molecular Chirality

Mateusz Pawlak[1,2], Aleksandra Wajda[1,3], Zofia Rejman[1], Maciej Roman[4], Tomasz P. Wróbel[4], Agnieszka Kaczor[1,*]

[1] Faculty of Chemistry, Jagiellonian University, 2 Gronostajowa Str., 30-387 Krakow, Poland.
[2] Doctoral School of Exact and Natural Sciences, Jagiellonian University, 11 Lojasiewicza Str., 30-348 Krakow, Poland.
[3] Laboratory for Biomedical Spectroscopic Applications (LBSA), Faculty of Pharmacy, Jagiellonian University Medical College, 9 Medyczna St., 30-688 Krakow, Poland.
[4] SOLARIS National Synchrotron Radiation Centre, Jagiellonian University, 98 Czerwone Maki Str., 30-392, Krakow, Poland.

[*]corresponding author's e-mail: agnieszka.kaczor@uj.edu.pl

## Content

**Fig. S1.** ECD and electronic absorption spectra of air-dried or lyophilized C-Dots and cysteine.

**Fig. S2.** VCD and infrared spectra of air-dried and lyophilized C-Dots and cysteine.

**Fig. S3**. Representative AFM and corresponding *s*-SNOM images (1400 cm$^{-1}$) of as-synthesized, lyophilized and resuspended in water or air-dried and resuspended in water C-Dots.

**Fig. S4**. Representative TEM images of C-Dots sonicated for 15 minutes and without sonication.

**Fig. S5**. ATR-IR spectra of solid C-Dots and cysteine.

**Fig. S6.** Electronic absorption spectra of air dried C-Dots.

**Fig. S7.** Fluorescence spectra of air-dried C-Dots.

**Fig. S8.** VCD and IR spectra of C-Dots post-functionalized with *L*/*D*-cysteine, recorded after complete conversion of substrates into the C-dots-cysteine assembly. Time-dependent changes in VCD and IR signals for the *L*-C-Dots and *D*-cysteine system. VCD and IR spectra of the *L*-C-Dots and *D*-cysteine system in the presence of dithiothreitol (DTT).

**Fig. S9.** VCD and IR spectra of C-Dots with *D*-cysteine and with *L*-cysteine, recorded after complete conversion of the substrates into the corresponding systems.

**Fig. S10.** ECD and electronic absorption spectra of *D*-cysteine-derived C-Dots and *L*-cysteine-derived C-Dots, recorded after complete conversion of the substrates into the corresponding complexes.

**Fig. S11.** VCD and IR spectra of *L*-C-Dots combined with *L*-phenylalanine, *L*-alanine, *L*-serine, and *D*-tyrosine, recorded approximately 4 hours after sample preparation.

**Fig. S12.** VCD and IR (**A-B**) spectra of *L*-C-Dots combined with *L*-homocysteine, recorded between 40 minutes and 4 hours after sample preparation.

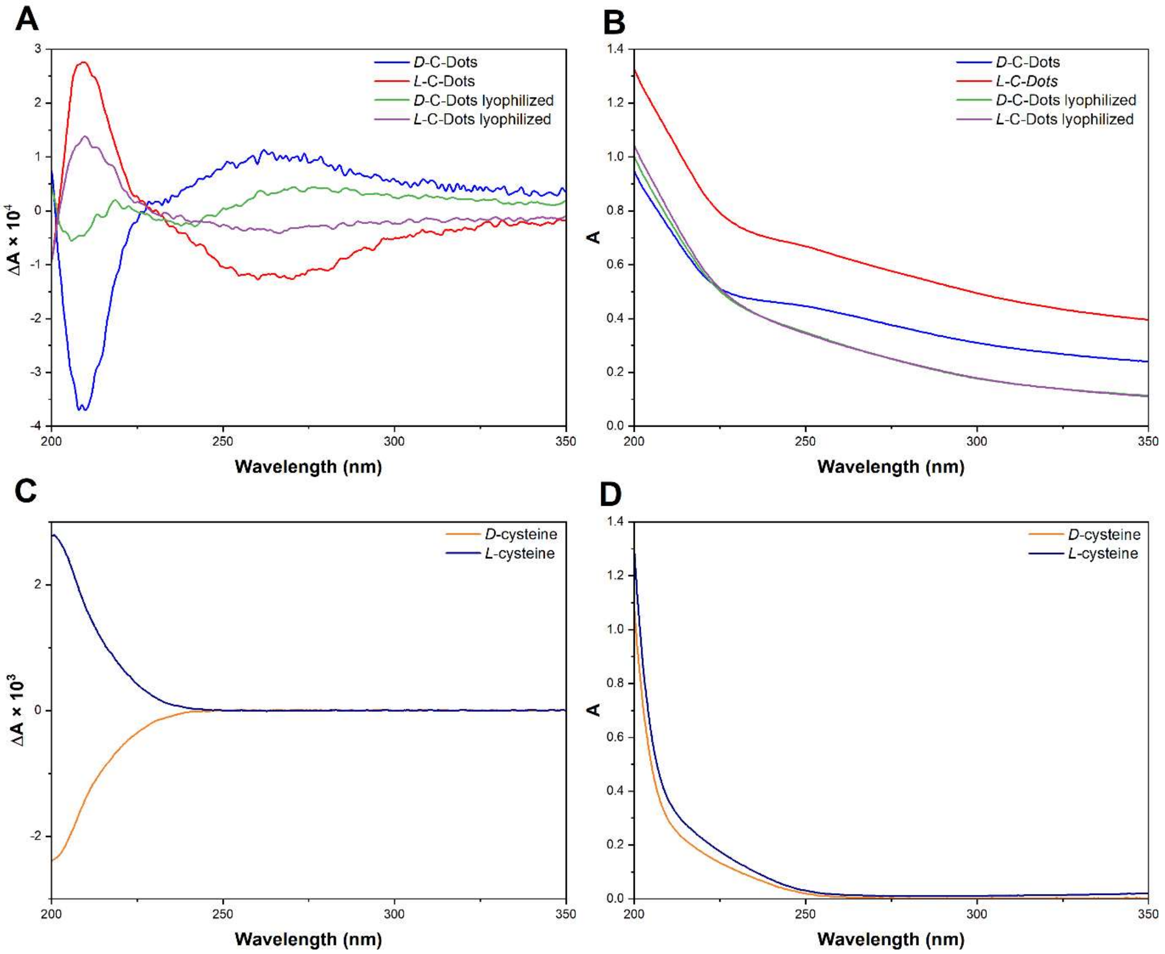


**Fig. S1.** ECD and electronic absorption spectra of air-dried or lyophilized C-Dots (**A**-**B**) and cysteine (**C**-**D**).

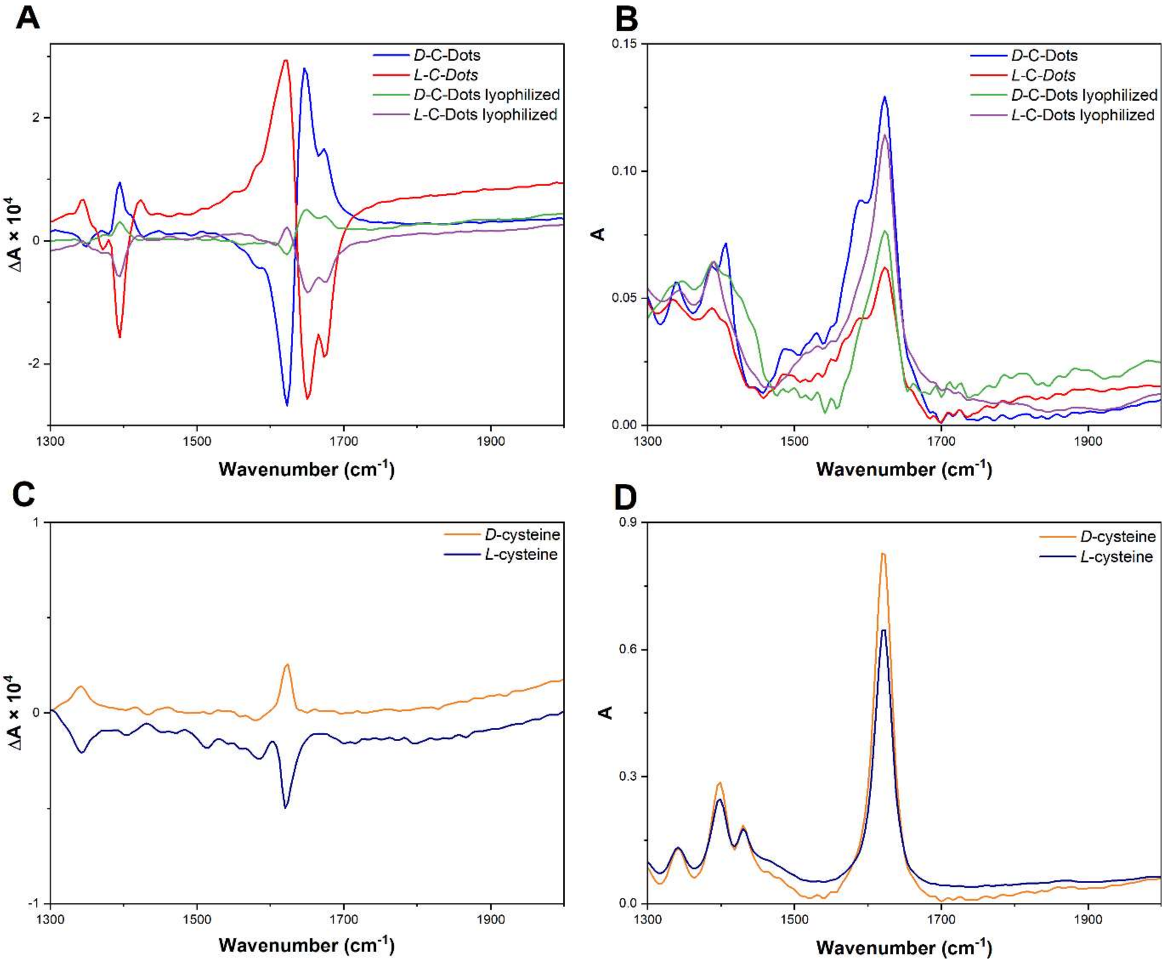


**Fig. S2.** VCD and infrared spectra of air dried and lyophilized C-Dots (**A-B**) and cysteine (**C-D**).

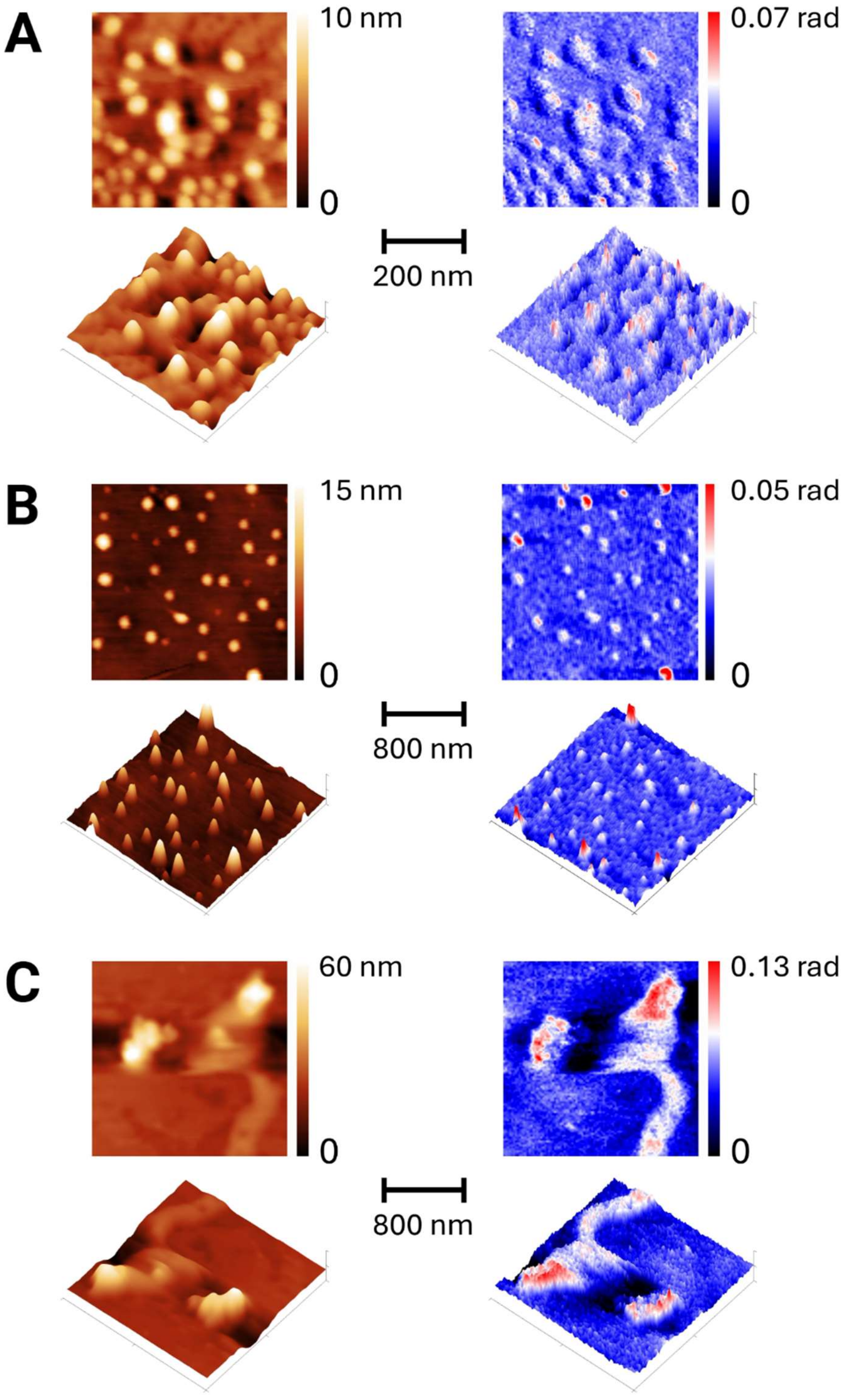


**Fig. S3**. Representative AFM and corresponding *s*-SNOM images (1400 cm$^{-1}$) of as-synthesized (**A**), lyophilized and resuspended in water (**B**) or air-dried and resuspended in water (**C**) C-Dots drop-cast onto a silicon substrate.

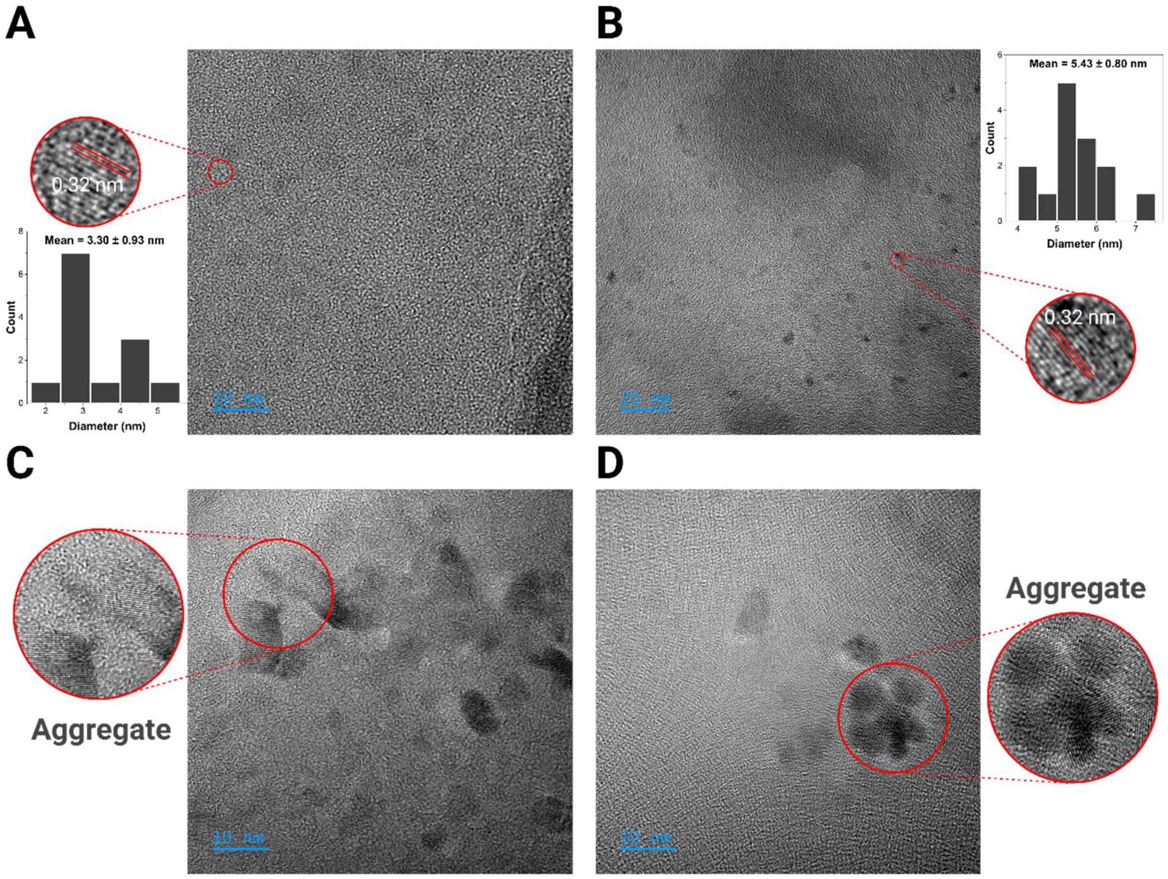


**Fig. S4**. Representative TEM images of air-dried and resuspended in water C-Dots sonicated for 15 minutes (**A, C**) and without sonication (**B, D**). The images demonstrate that C-Dots exist in the form of non-aggregated (**A**, **B**) and aggregated particles (**C**, **D**). Size distribution analysis for monomeric C-Dots (**A**, **B**). Monomers show typical size and morphology with the distance between graphene layers of 0.32 nm.

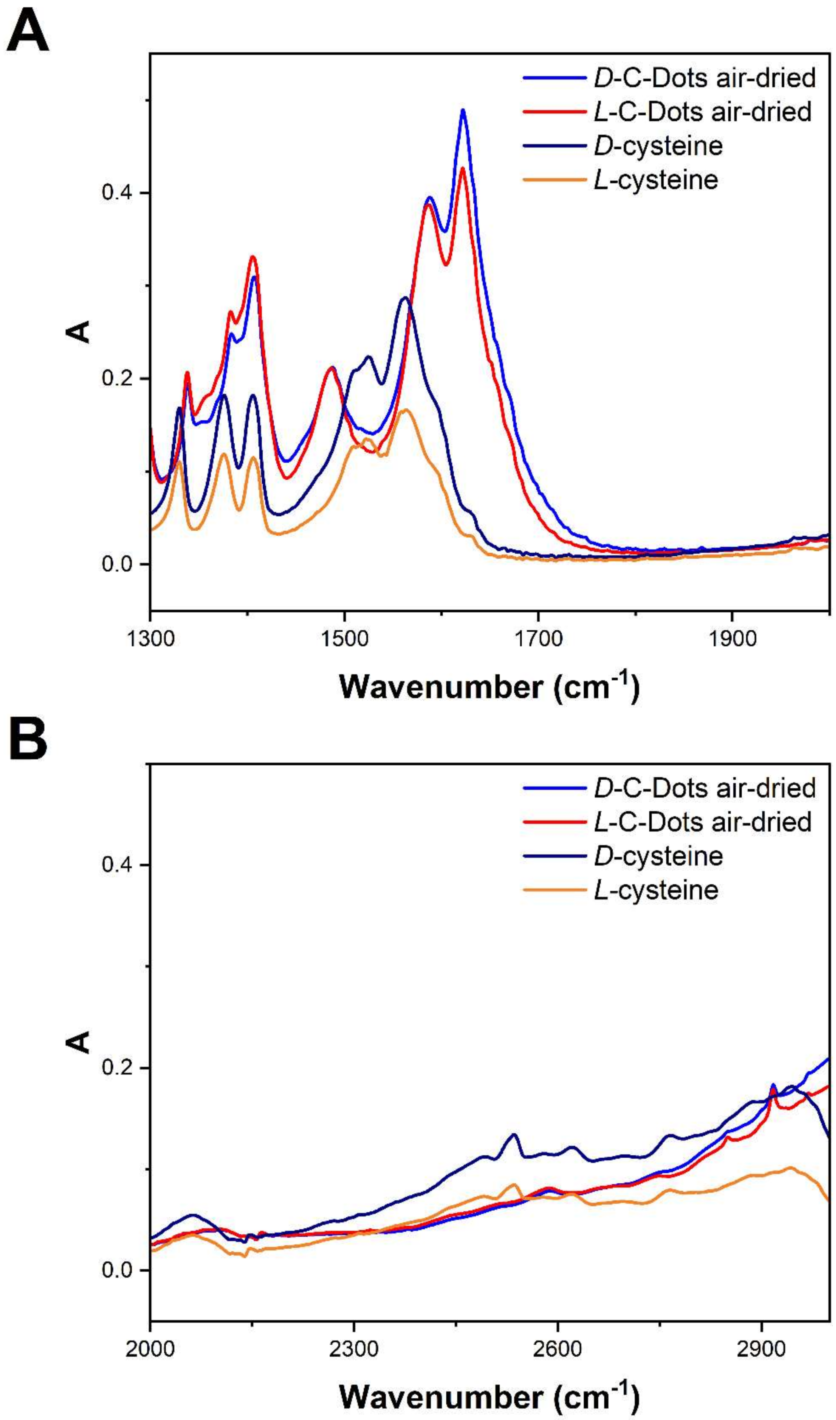


**Fig. S5**. ATR-IR spectra of solid C-Dots and cysteine in the 1300–2000 cm$^{-1}$ range (**A**), and in the high-wavenumber region showing bands assigned to–S-H stretching vibrations at 2550–2600 cm$^{-1}$ and -N-$H_2$ stretching vibrations at ca. 3100-3300 cm$^{-1}$ (**B**).

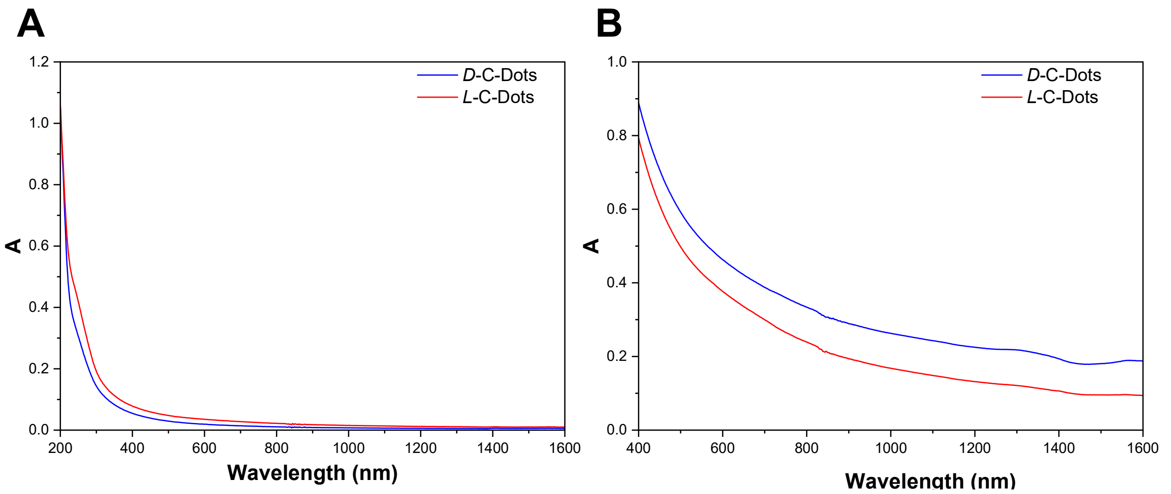


**Fig. S6.** Absorbance spectra of air-dried *L*/*D*-C-Dots: (**A**) 0.1 mg/mL in the range 200–1600 nm; (**B**) 1 mg/mL in the range 380–1600 nm.

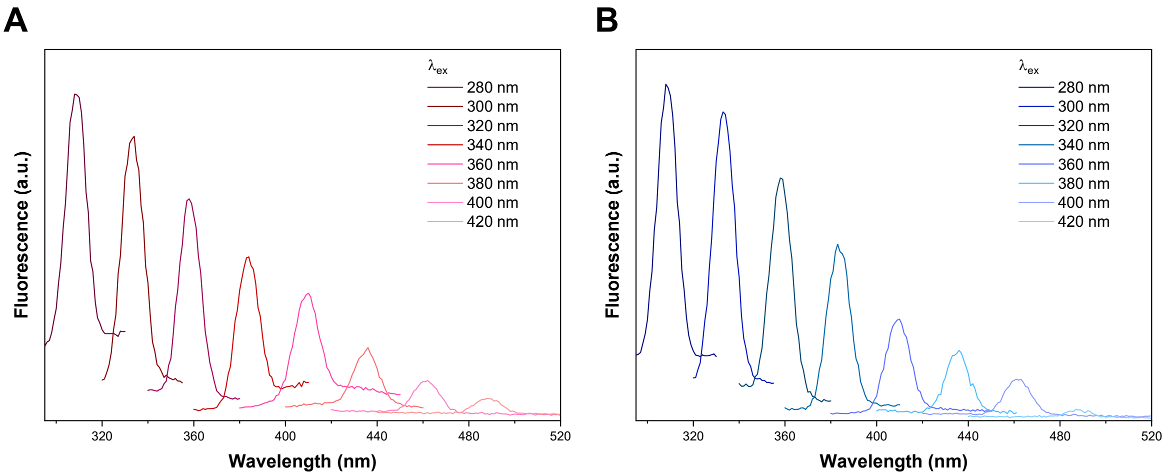


**Fig. S7.** Fluorescence spectra of *L*-C-Dots: (**A**) and *D*-C-Dots (**B**) at the concentration of $10^{-4}$ mg/mL in the range 300-520 nm.

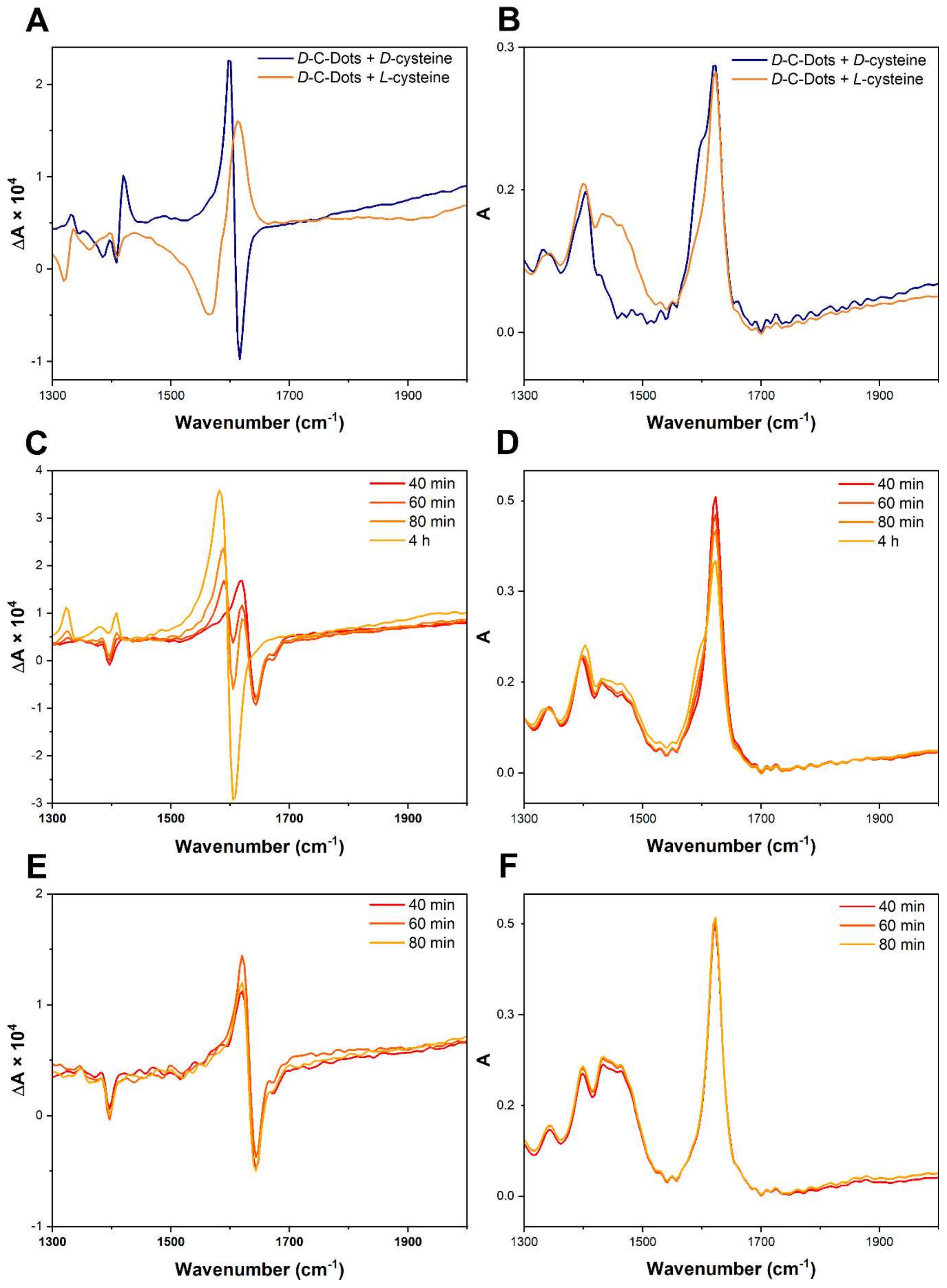


**Fig. S8.** VCD and IR spectra of C-Dots post-functionalized with *L*/*D*-cysteine recorded after complete conversion of substrates into the C-Dots-cysteine assembly (**A**–**B**). Time-dependent changes in VCD and IR signals for the *L*-C-Dots and *D*-cysteine system (**C**–**D**). VCD and IR spectra of the *L*-C-Dots and *D*-cysteine system in the presence of dithiothreitol (DTT) (**E**–**F**).

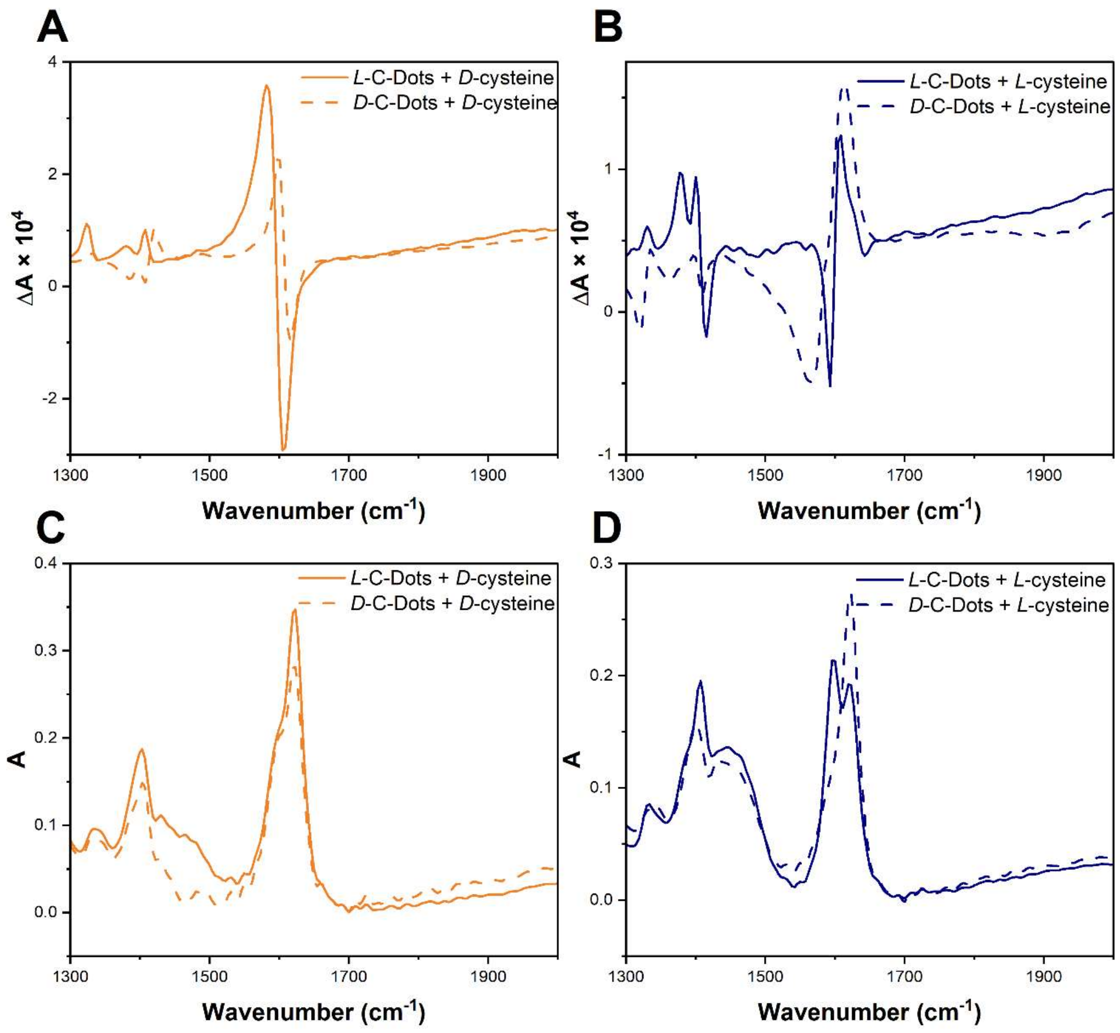


**Fig. S9.** VCD and IR spectra of C-Dots with *D*-cysteine (**A**, **C**) and with *L*-cysteine (**B**, **D**), recorded after complete conversion of the substrates into the corresponding systems.

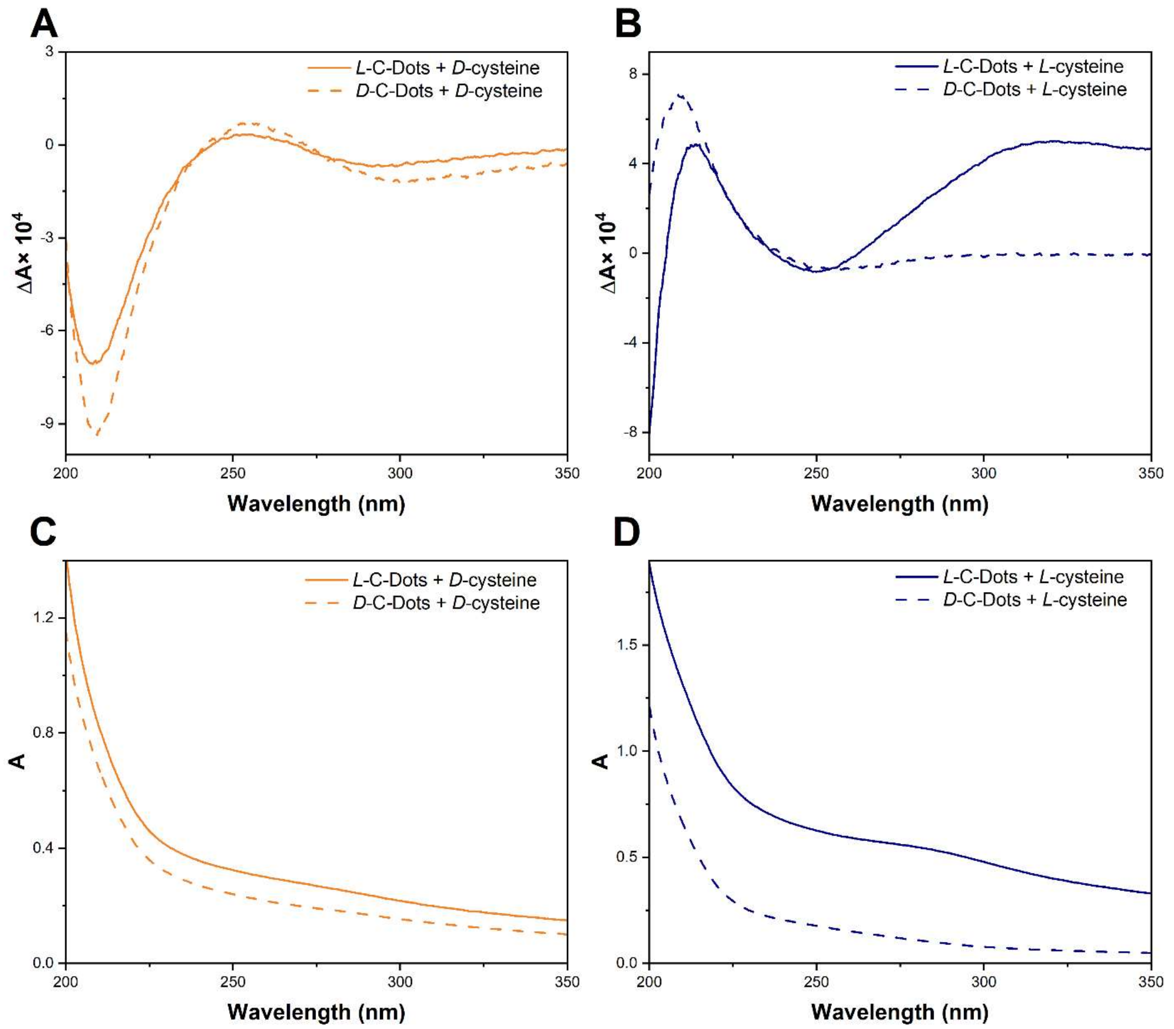


**Fig. S10.** ECD and electronic absorption spectra of *D*-cysteine-derived C-Dots (**A**, **C**) and *L*-cysteine-derived C-Dots (**B**, **D**), recorded after complete conversion of the substrates into the corresponding complexes.

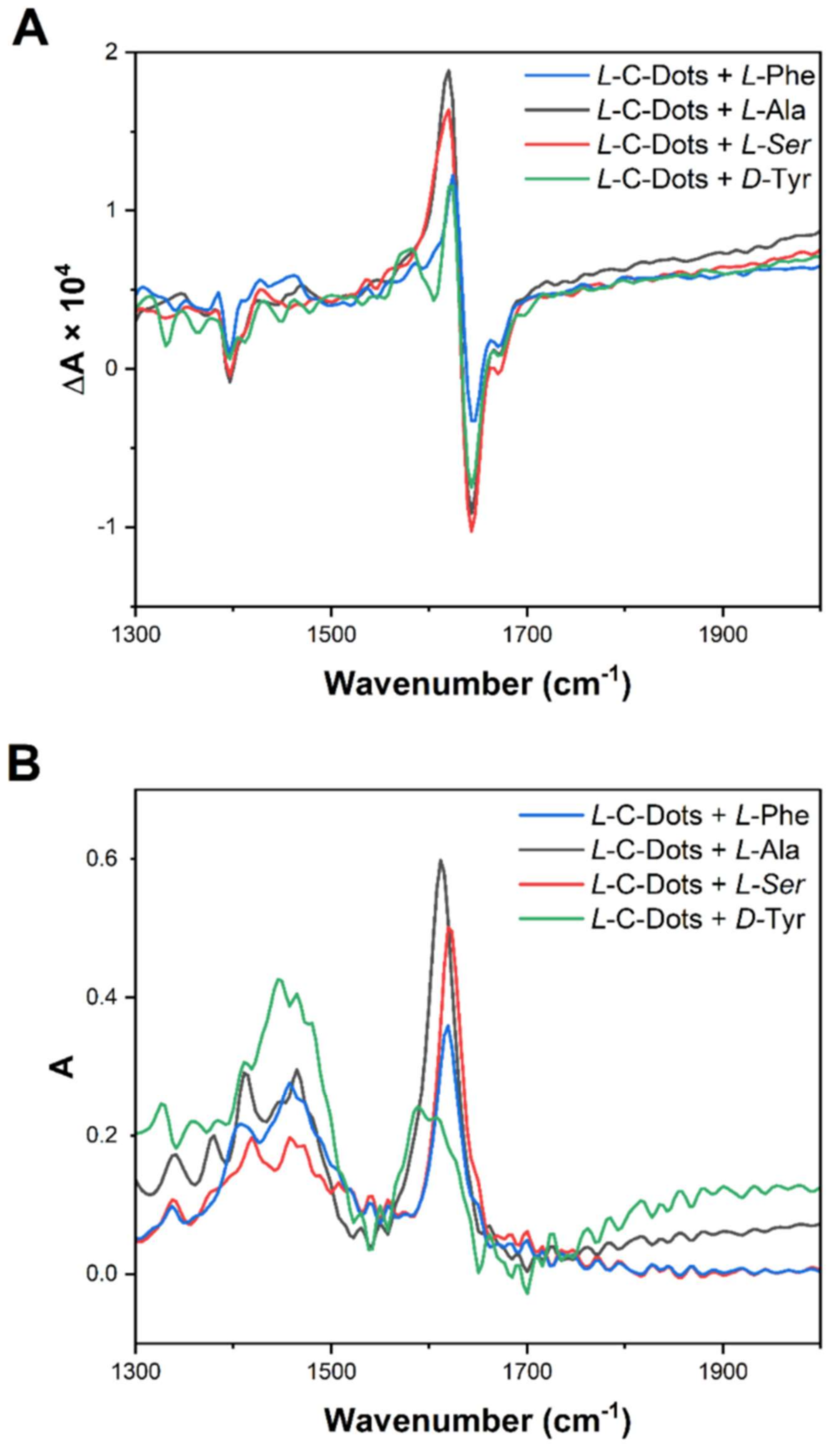


**Fig. S11.** VCD spectra (**A**) and IR spectra (**B**) of *L*-C-Dots combined with *L*-phenylalanine, *L*-alanine, *L*-serine, and *D*-tyrosine, recorded approximately 4 hours after sample preparation.

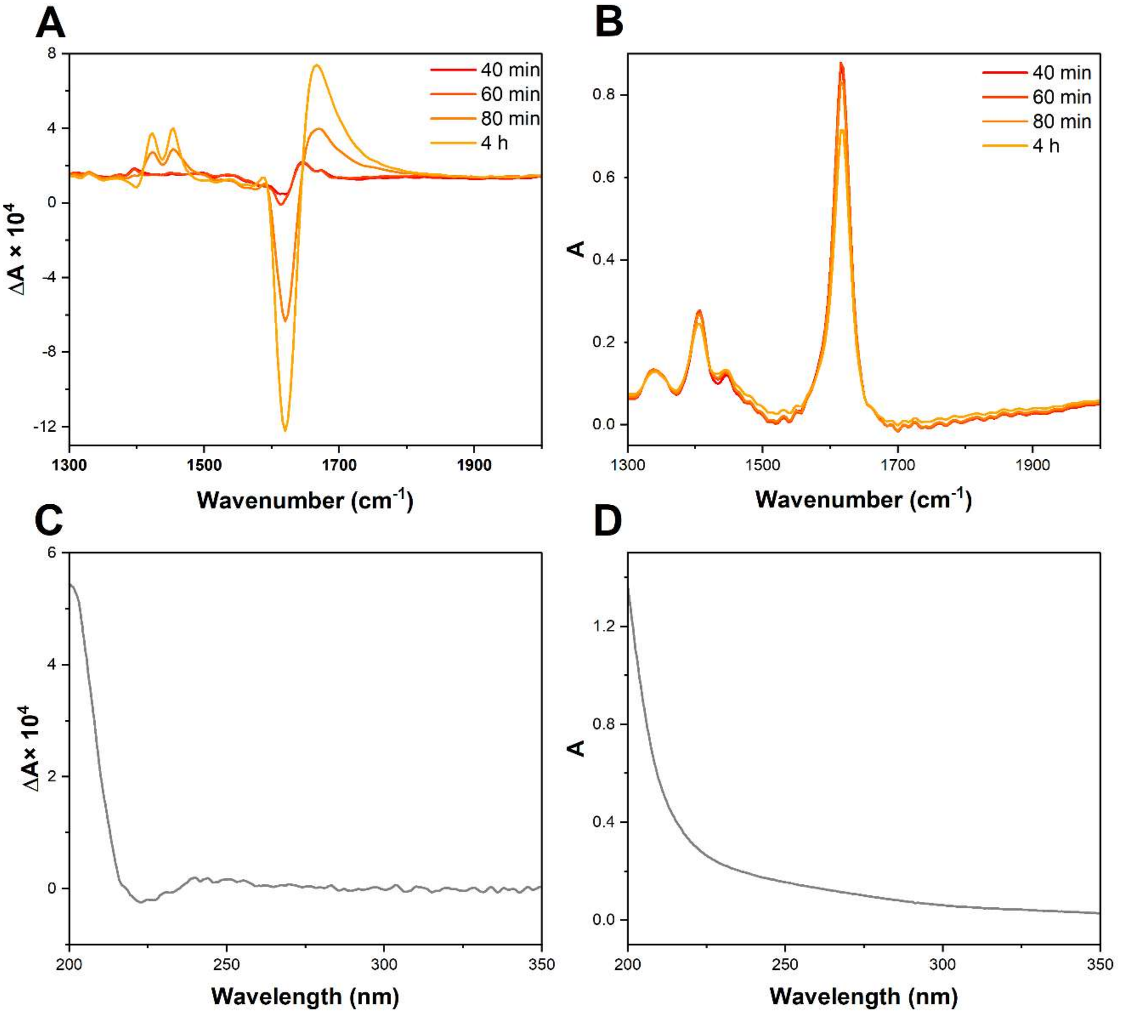


**Fig. S12.** VCD and IR (**A**-**B**) spectra of *L*-C-Dots combined with *L*-homocysteine, recorded between 40 minutes and 4 hours after sample preparation. ECD and electronic absorption (**C**-**D**) spectra recorded after complete conversion of the substrates into the corresponding complex.
